# Evaluation of Assembly Strategies Using RNA-Seq Data Associated with Grain Development of Wheat (*Triticum aestivum* L.)

**DOI:** 10.1371/journal.pone.0083530

**Published:** 2013-12-12

**Authors:** Huai-Zhu Li, Xiang Gao, Xiao-Yan Li, Qi-Jiao Chen, Jian Dong, Wan-Chun Zhao

**Affiliations:** 1 Department of Seed Science, College of Agronomy, Northwest A&F University, Yangling, Shaanxi Province, People's Republic of China; 2 Wheat Engineering Research Center of Shaanxi Province, Yangling, Shaanxi Province, People's Republic of China; Harbin Institute of Technology, China

## Abstract

Wheat (*Triticum aestivum* L.) is one of the most important crops cultivated worldwide. Identifying the complete transcriptome of wheat grain could serve as foundation for further study of wheat seed development. However, the relatively large size and the polyploid complexity of the genome have been substantial barriers to molecular genetics and transcriptome analysis of wheat. Alternatively, RNA sequencing has provided some useful information about wheat genes. However, because of the large number of short reads generated by RNA sequencing, factors that are crucial to transcriptome assembly, including software, candidate parameters and assembly strategies, need to be optimized and evaluated for wheat data. In the present study, four cDNA libraries associated with wheat grain development were constructed and sequenced. A total of 14.17 Gb of high-quality reads were obtained and used to assess different assembly strategies. The most successful approach was to filter the reads with Q30 prior to *de novo* assembly using Trinity, merge the assembled contigs with genes available in wheat cDNA reference data sets, and combine the resulting assembly with an assembly from a reference-based strategy. Using this approach, a relatively accurate and nearly complete transcriptome associated with wheat grain development was obtained, suggesting that this is an effective strategy for generation of a high-quality transcriptome from RNA sequencing data.

## Introduction

Wheat (*Triticum aestivum* L.) is one of the most widely cultivated crops because of its high yield and nutritional value [1], [2], [3].Wheat has a very large and complex genome (17Gb, 40 times larger than *Oryza sativa*), which contains three homoeologous genomes (2n = AABBDD; AA from *Triticum urartu*, BB from a species that is unknown but which maybe of the section Sitopsis,to which *Aegilops speltoides* belongs, and DD from *Aegilops Tauschii*). Whole genome sequencing of wheat has been considered too challenging because of the relatively large size, proportion of repeat sequences (∼80%, primarily retroelements) and number of paralogs and diverse alleles [1], [4]. To date, no well-established wheat genome sequence is available [5]. Transcriptome analysis could provide a better understanding of wheat genetics, however, the relatively high proportion of orthologs, paralogs and isoforms in wheat and the relatively low levels of gene expression also presents several unique challenges for transcriptome research [6]. RNA sequencing (RNA-seq) with its unprecedented sensitivity and accuracy [7], [8], [9] has been widely used for transcript profiling, Single nucleotide polymorphism (SNP) detection and differentially expressed gene analysis [10], [11], [12], [13], [14], [15]. Reads obtained using RNA-seq are often 35–500 bp, shorter than traditional expressed sequence tag by Sanger sequencing technologies [7], [8]. Therefore, it is necessary to reconstruct full-length transcripts from these short reads [16]. The short read assembly programs, SOAPdenovo-Trans [17], Trans-ABySS [18], [19], Velvet-Oases [20], [21] and Trinity [22] have been successfully applied to assemble transcriptomes in many organisms [10], [22], [23], [24], with both the single *k*-mer (SK) and multiple *k*-mer (MK, not for Trinity) methods. Previous studies suggested that the specific characteristics of the different programs and strategies could greatly affect the assembly [22], [25], [26], [27]. Of the four programs, Trinity was the most efficient and sensitive in assembling full-length transcripts and isoforms in several model organisms [22], and it with the SK strategy outperformed Trans-ABySS with the MK strategy in assembling a hexaploid wheat transcriptome [6].

Reference-based assembly strategies are often applied to reconstruct a transcriptome for which a reference genome is available [6], [16]. However, this strategy is impractical for wheat, which lacks a well-characterized genome. Fortunately, draft genomes of the wheat A and D-genome progenitors were recently established [28], [29], which may serve as a good reference for wheat short read assembly. On the other hand, *de novo* assembly of short reads into a transcriptome can identify all transcripts, separate isoforms, and reconstruct full–length transcripts. However, *de novo* transcriptome assembly requires a much higher sequencing depth and ideal hardware than the reference-based strategy for the same task. Furthermore, *de novo* transcriptome assembly programs are very sensitive to sequencing errors and fail to distinguish highly similar transcripts (for example, alleles or paralogs) [16]. These observations suggested that a combinationof reference-based and *de novo* strategies would be a superior approach that warranted testing in wheat.

In the present study, sequence reads associated with grain development of wheat were obtained using RNA-seq. To reconstruct an accurate and nearly complete transcriptome, several factors affecting read assembly were evaluated, including k-mer values, programs (SOAPdenovo, Trans-ABySS, Velvet-Oases and Trinity), methods (SK or MK) and overall assembly strategies were evaluated. Determining the best strategy for wheat transcriptome assembly from RNA-seq data could provide a crucial guideline for reconstruction of high quality transcriptomes from complex genomes. In addition, the reconstructed transcriptome from this study will be useful for future expression profiling and differential expression analysis of genes associated with wheat grain development.

## Materials and Methods

### Plant materials and sampling

The common wheat variety P271 was cultured during the wheat growing season (October to June) under natural conditions in Yangling, Shaanxi province (34.26°N, 108.14°E), fertilized with urea (60 kg/ha) and watered periodically. The mainstem ears were tagged on the morning when the anthers first appeared outside the florets of the spikelets. The labeled spikelets were harvested at 4, 8 and 12 days after pollination (DAP4, DAP8 and DAP12). Developing grains were collected from the first florets of the four central spikelets. The embryo of each grain was removed and the remaining endosperm and seed coat were designated as EDAP4, EDAP8 and EDAP12, respectively. Each group at this stage consisted of at least 200 seeds from 30 spikes, which were immediately frozen in liquid nitrogen. All materials were stored at −80°C until RNA extraction [30].

### RNA isolation and library preparation

Total RNA samples from the three sample groups (EDAP4, EDAP8 and EDAP12) were isolated using the Trizol reagent (Invitrogen) and then treated with *Dna*se I (Promega) at a concentration of 1 U/µg. The Mix-RNA sample was composed of equal amounts of RNA from EDAP4, EDAP8 and EDAP12. The quality and quantity of the RNA samples were examined using the Agilent 2100 Bioanalyzer (Agilent Technologies). The RNA samples were then purified using the TruSeq RNA Sample Preparation Kit (Illunima). Briefly, the poly-A mRNA was purified from 10 µg of total RNA using poly-T oligo-attached magnetic beads. The mRNA was fragmented using divalent cations at 95°C. The fragmented RNA was used for the first and second strand cDNA synthesis. The cDNA fragments were end-repaired and ligated to adapters for PCR purification and enrichment to create the final cDNA libraries. The number of PCR cycles was minimized to avoid amplification bias. Fragments from 250 to 350 bp were selected by agarose gel purification to produce the libraries for cluster generation and sequencing. Paired-end sequencing of the four cDNA libraries (EDAP4, EDAP8, EDAP12 and Mix) was performed using the Illumina sequencing platform (GAII).

### Raw reads filtering

Paired-end (PE) raw reads from the four libraries (EDAP4,EDAP8,EDAP12 and Mix) were trimmed using the NGSQCToolkit (version 2.2.3) without primer/adaptor filtering, with a read length cutoff of 50% and with a base Phred quality scores threshold of Q30 (*P*-value≤0.001) [31]. The low-quality base calls at the ends of each read were filtered and reads that were <50 bp or singletons were discarded to generate the final high quality (HQ) read libraries. The data were deposited into SRA archive with accession number SRP029372.

### *De novo* assemble with four assemblers

To evaluate the performance of the four assembly programs, all of the four read libraries HQ reads were *de novo* assembled using SOAPdenovo-Trans (release 1.01) with average insert size  =  300 bp [17], Trans-ABySS (version 1.3.2) [19], Velvet (version 1.2.07) with library insert length  =  300 and minimum contig length  =  100 [20], Oases (version 0.2.08) [21], Trinity (release 20120608) with minimum contig length  =  100 [22]. Similar assembly parameters were adopted in the four programs.

The k-mer length (k) is one of the most important parameters because it defines the sequence overlap between two reads forming a contig and can substantively affect the final assembly product [19]. Shorter k values tend to be better for less expressed transcripts, whereas larger k values are more practical for highly expressed sequences [20], [32]. A single k-mer value is therefore unlikely to yield an optimal overall assembly. Alternatively, compiling assemblies with multiple k-mer values improves accuracy, sensitivity, and specificity of the overall *de novo* transcriptome assembly [19], [32].

**Figure 1:**
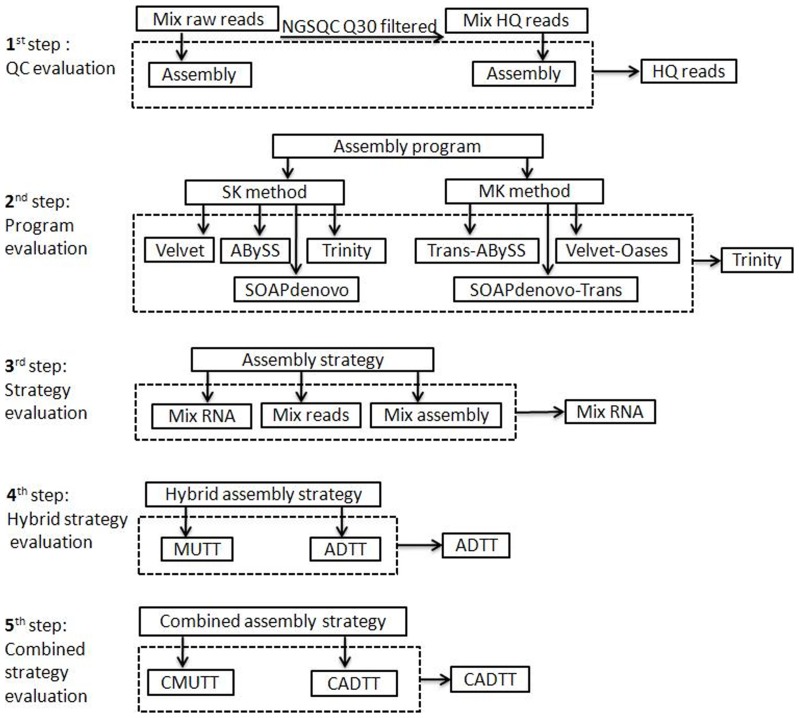
Analysis process flow diagram. This analysis process mainly consisted of four steps: (1) evaluation of raw and HQ read assembly, with HQ reads chosen as input for the next step; (2) evaluation of the performance of four assembler programs using SK and MK methods, with Trinity selected for downstream analysis; (3) evaluation of different data volume assembly strategy; and (4) evaluation of two hybrid assembly strategies; (5) evaluation of two combined assembly strategy.

SK and MK approaches were adopted in the SOAPdenovo-Trans, Trans-ABySS and Velvet-Oases assemblies. SK length ranged from 25 to 97 bp with a length interval of 6. Only the SK approach with k of 25 bp was used in the Trinity assembly. For the MK methods, Trans-ABySS merged all of the SK assemblies in the first step of the analysis pipeline. Oases merged all of the Velvet SK assemblies using an array of hash lengths and a dynamic noise filtering process [21]. SOAPdenovo-Trans merged all the SK assemblies and then removed redundancy. For each assembly program, the performance at different SK lengths was compared, and the k-mer value of the best assembly was selected. Next, the best SK assembly of each program was compared to the MK assembly for that program (Figure 1). To examine overlap/similarity among the seven assemblies, pairwise alignment was performed using the Basic Local Alignment Search Tool like (BLAST-Like) Alignment Tool (BLAT) [33]. Similarity was defined as the percentage of the two assemblies with >95% identity and >80% overlap.

All the assemblies were performed on a Linux server with an 80-core processor and 512 GB of memory. HQ transcripts over 300 bp were used for the downstream analysis.

### Assembly statistics metrics calculation

To compare the assemblies generated by different programs and methods, assembly statistics were calculated using the N50Stat.pl tool of the NGSQCToolkit [31]. Statistics metrics used in this study included the total number of sequences≥300bp, the average, median, and N50 sequence lengths, the guanine-cytosine (GC) content and the proportion of ambiguous base calls (Ns). N50 was defined as the contig length where 50% of the assembly was represented by contigs of this size or longer [34].

### Mapping reads back to transcripts

Mapping reads back to transcripts is one of the most important methods to assess assembly quality. To obtain reads mapped back to transcript (RMBT) values for each assembly, Bowtie (version 0.12.7), with default parameters [35], was applied to map PE short reads back to the assembled contigs. The mapping metrics included reads that aligned concordantly exactly 1 times (ACE1), reads that aligned concordantly >1 times (AC>1), and the overall alignment rate (OAR).

### Mapping assembly transcripts to reference genes

Criteria to comprehensively assess transcriptome assemblies has been difficult to establish and is still in development. Recent studies have reported several important evaluative metrics for both simple and complex transcriptomes, including completeness (the degree to which the references gene is covered by the assembled contigs), contiguity (the likelihood that a full-length transcript is represented as a single contig) and accuracy (a measure of the correctness of an assembly) [16], [36]. However, it is difficult to assess assembly quality by these metrics without a set of well-established expressed reference transcripts that, ideally, includes transcripts of varying length and expression levels [16]. For our purposes, a full-length common wheat cDNA data set was available (TriFLDB-6137, http://trifldb.psc.riken.jp/download.pl) with perfect replacement for a reference transcript that contains 6137 sequences ranging from 131 to 8930 bp (N50 length  = 1996 bp and average sequence length  = 1753 bp). The fact that the data set contains many long transcripts makes it particularly useful in estimating contiguity and completeness [37]. This, and another reference dataset (TriallCDNA) containing 97481 sequences ranging from 100 to 10382 bp, representing nearly all wheat genes [4], were used to assess the completeness, contiguity and accuracy of each of our assemblies. BLAT [33] with default parameters was applied to map each assembly to the reference sequences [16], [36]. Completeness is the percentage of reference sequences for which >80% of the length is covered by assembled contigs. Contiguity is the percentage of complete reference sequences for which >80% of the length is covered by a single assembled transcript. Accuracy is the percentage of the assembly that shares ≥95% identity with the reference sequences. Perfect accuracy is the reference sequences for which >80% of the length is covered by a single assembled transcript and shared ≥95% identity with the transcript.

To assess the new transcripts detection capability of each assembly program, we aligned each assembly to the reference sequences using BLAST with the requirement of >100 bp match. The number and percentage of assembled contigs that aligned to the reference sequences with identity ≥95% or ≥70% were used for assessment.

### Different data assembly strategies

Reconstruction of a comprehensive transcriptome from short reads involves many complex assembly issues. This study compared several different data assembly strategies including mixing of RNA samples (EDAP4, EDAP8, EDAP12, Mix-RNA), merging of reads from different libraries (EDAP4, EDAP8, EDAP12, Mix-reads) and merging of single library assemblies then removal of redundancies (Mix-assembly). The quality of the output from the different strategies was measured by the total number of sequences obtained, the average length of reads, the N50 length, and the ACE1, AC>1, and OAR values).

### Hybrid assembly with wheat cDNA

TriallCDNA data was used to improve assembly quality [4]. HQ reads were mapped to TriallCDNA using Bowtie2 with default parameters [37]. The sequences of TriallCDNA which were mapped at least once by the HQ reads, were extracted and used to design the MappedTriallCDNA data set. The unmapped HQ reads were *de novo* assembled usingTrinity and then merged with MappedTriallCDNA using CD-HIT-EST [38] with 100% identity to generate an overall assembly. This strategy is termed merging unmapped transcripts with TriallCDNA (MUTT**)**. Another strategy was to firstly *de novo* assemble the HQ reads, then assemble the resulting transcripts with MappedTriallCDNA using CAP3 [39] with 50 bp overlat and share at least 98% identity. We termed this strategy assemble *de novo* transcripts with TriallCDNA (ADTT).

### Combined strategies

To create an even more comprehensive transcriptome, we next combined the reference-based and *de novo* assembly approaches. This strategy brings together the high sensitivity of reference-based assembly with the greater ability of *de novo* assembly to detect novel transcripts [16]. Firstly, the HQ reads from the four libraries (EDAP4, EDAP8, EDAP12 and Mix) were assembled by Cufflinks [40] using the draft A- and D-progenitor genomes as references [28], [29], [40]. The assemblies were then combined with the MUTT or ADTT assembly by CD-HIT-EST [38] with 95% identity to generate overall assemblies. These strategies termed combined reference-based assembly with MUTT assembly (CMUTT) and combined reference-based assembly with ADTT assembly (CADTT), respectively.

### Final transcriptome assembly and annotation

Through a series of analyses, the best strategy was selected to assemble the wheat grain development transcriptome. The contigs from the EDAP4, EDAP8, EDAP12 and Mix libraries assembled by CADTT strategy were merged and clustered by CD-HIT-EST with identity of 95%. To obtain annotation information about the assembled transcript, the final transcriptome was aligned by BLAST searched against the non-redundant protein sequence database with default parameters.

### Statistical analysis

All statistical analyses were performed by Excle (Microsoft). An α value of 0.05 was used as the criterion for statistical significance

## Results

### Overview of analysis

Raw RNA-seq data and Q30 filtered HQ data of four wheat developmental libraries were firstly *de novo* assembled using the Trinity assembler to assess the Q30 quality control effect on assembly. Then four assembler programs and several different assembly strategies were evaluated and compared using these data to select the most accurate and comprehensive approach to re-construct the wheat transcriptome (Figure 1). Assembly evaluation metrics included descriptive statistics, RMBT, novel gene detection capability and computing resource usage. The raw and HQ reads (Q30 filtered) from the four libraries were statistically analyzed separately. However, since there were large differences in sequencing depth among the four libraries, and it has a profound impact on assembly quality statistical analysis, the paired t-test method was used instead of analysis of variance (ANOVA) to assess the effect of Q30 on assembly quality. For the same reason, two-way ANOVA without replication was applied in the other statistical analyses.

### Effect of Q30 on assembly quality

Four cDNA libraries (EDAP4, EDAP8, EDAP12 and Mix) associated with wheat grain development were constructed for PE sequencing. A total of 88.145 million 100 bp PE raw reads, representing 17.63 Gb, were generated (Table 1). After filtering of low-quality bases (quality score<Q30), short reads (<50 bp) and singletons, 70.864 million HQ PE reads remained for downstream analyses. The proportion of HQ reads from the four libraries varied from 77.22 to 83.35%, with an average of 79.19%.

**Table 1:** Statistics of raw and HQ reads.

Metrics	EDAP4	EDAP8	EDAP12	Mix	Total	Average
Number of raw reads	24863171	11317131	11964057	40001060	88145419	
GC content (%)	41.83	46.23	45.39	53.1		46.6375
Gb	13.2	6	6.4	22	47.6	
Number of HQ^a^ reads	19467396	8815361	9239158	33342047	70863962	
GC content (%)	41.22	45.49	44.81	52.63		45.18
Gb	10.4	4.8	5	17.8	38	
HQ reads proportion	78.30%	77.89%	77.22%	83.35%		79.19%

^a^ Q30 filtered reads.

Raw and HQ reads of the four libraries were *de novo* assembled using Trinity [22] to assess the effect of sequencing errors on assembly quality. T-test analysis indicated that the number of contigs ≥500bp) assembled from the HQ reads was significantly lower (*P*-value<0.05) than from the raw reads. However, the N50 and average sequence lengths were slightly higher for the HQ read assembly (Table 2), indicating that the Q30 quality control results in longer and fewer transcripts.

**Table 2:** Statistics of the Mix raw and HQ read assemblies.

Metrics	MeanD-value^a^	Sd^b^	t Stat	*P*-value	Sig
Total reads	−4320364	2039492	−4.24	<0.05	*
Total sequences≥300 bp	−2742	2063	−2.66	>0.05	
Total sequences≥500 bp	−1235	670	−3.69	<0.05	*
Total bases	–1521587	697164	–4.37	<0.05	*
Average sequence length	10.66	25.28	0.84	>0.05	
N50 length	22.75	47.32	0.96	>0.05	
GC content OR (G + C)s	–0.01	0.00	–3.45	<0.05	*

^a^ The difference between the raw and HQ read assemblies.

^b^ Standard deviation.

% level. Mean significant at 5

**Figure 2:**
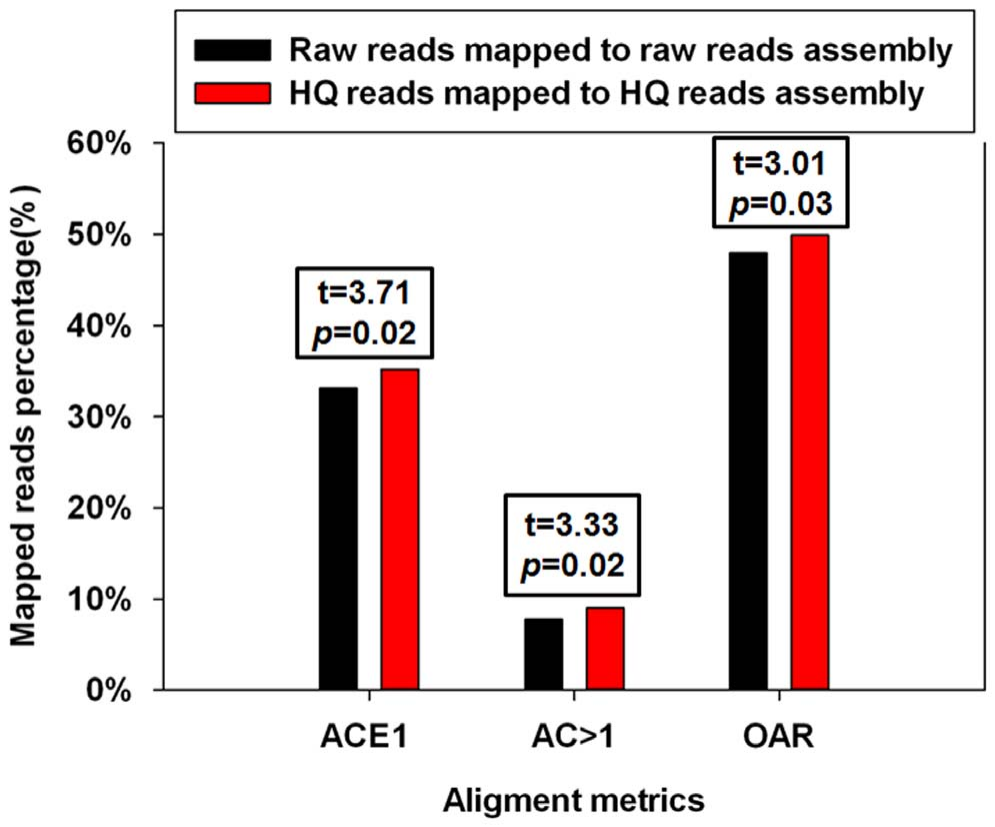
Comparison of raw and HQ read assembly by mapping reads back to assembled contigs. Raw and HQ reads were mapped back to the raw readand HQ read assemblies (contigs >300 bp), respectively. The assessment metrics included ACE1 and AC>1 percentagesandOAR. Statistical analysis was performed using paired t-tests and *P*-valuesrefer todifferences between the two assemblies.

RMBT results indicated that the Q30 quality control improved assembly quality and mapping percentage. The total number of HQ reads was 19.61% lower than raw reads, and the percentages of reads that could be mapped back to the assembled contigs was higher for the HQ reads, with ACE1, AC>1 and OAR 2.09, 1.22 and 1.96% higher for HQ than raw reads, respectively (Figure 2, Table S1).

Assemblies from the raw and HQ reads were separately aligned against the wheat reference databases TriFLDB-6137 and TriallCDNA, using BLAT [33]. The completeness and contiguity of the HQ read assemblies were lower than that for the raw read assemblies. In contrast, the accuracy of the HQ read assemblies was much higher than the raw read assemblies, especially when using TriallCDNA as the reference (Table 3).

**Table 3:** Quality of assemblies mapped to wheat reference genes.

Metrics	Mean D-value^e^	Sd^f^	t Stat	*P*-value	Sig
Aligned to TriFLDB-6137					
Completeness^a^	−1.88%	0.01	−3.40	<0.05	*
Contiguity^b^	−1.41%	0.01	−3.64	<0.05	*
Perfect accuruacy^c^	−1.38%	0.01	−2.90	>0.05	
Accuruacy^d^	2.11%	0.01	3.86	<0.05	*
Aligned to TriallCDNA					
Completeness	−1.14%	0.01	−2.64	>0.05	
Contiguity	−1.03%	0.01	−3.40	<0.05	*
Perfect accuracy	0.03%	0.01	0.09	>0.05	
Accuracy	2.18%	0.01	4.30	<0.05	*

≥80% of the length).^a^ Percentage of reference sequences covered by the assemblies (

% of the length).^b^ Percentage of complete reference sequences covered by a single assembled contig (80

%) that share ≥95% identity with the contig.^c^ Percentages of reference sequences covered by a single assembled contig (80

≥95% identity with the reference sequences.^d^ Percentage of assembled contigs that share

^e^ The mean difference between the HQ and raw read assemblies.

^f^ Standard deviation.

% level. Mean significant at 5

### Assembly performance of programs with SK vs. Mk methods

**Figure 3:**
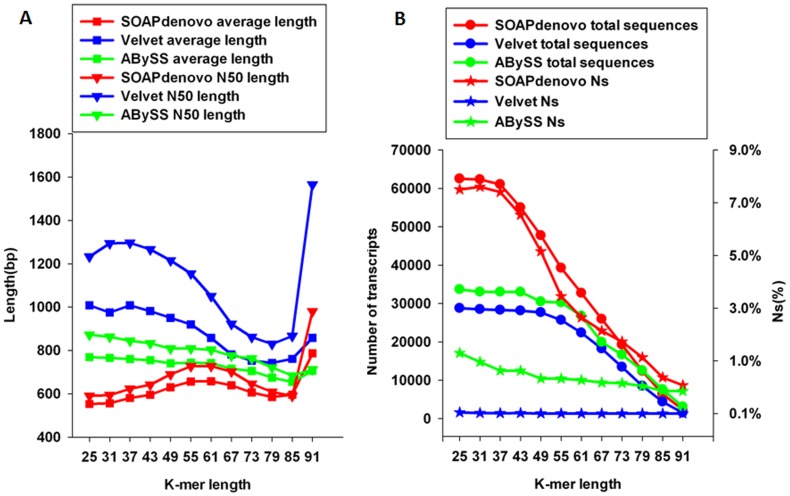
Performance of three programs using the SK method. The statistics of HQ read assembly by SOAPdenovo (red), Velvet (blue) and ABySS (green) with different single k-mer lengths (x axis). Assembly statistics metrics included the average contig length (squares), the N50 length (triangles), total sequences (circles) and percentage of Ns (stars).

The HQ reads from the four libraries were then *de novo* assembled using four different software programs and evaluated by assembly statistics. Two-way ANOVA showed that for some programs, as k increased from 25 to 91 bp, the average contig length and N50 length gradually increased, peaked at a k  =  61 bp for SOAPdenovo and at a k  =  37 bp for Velvet, and then dropped off sharply (Figure 3A, Tables S2 and S3). In contrast, the average and N50 lengths in the ABySS assembly firstly decreased with k increase and then increased slightly. The corresponding SK peaks of each value metric were different, ranging from k of 25 to 61bp (Figure 3A, Table S4). The total number of sequences decreased from 62515 (at k  =  43) to 280 (at k  =  97), from 28792 (at k  =  31) to 164 (at k  =  97), and from 33698 (at k  =  25) to 1070 (at k  =  97) in the SOAPdenovo, Velvet and ABySS assemblies, respectively (Tables S2 and S4). The percentage of Ns decreased with increasing k in all three programs(Figure 3, Tables S2, S3 and S4).

**Figure 4:**
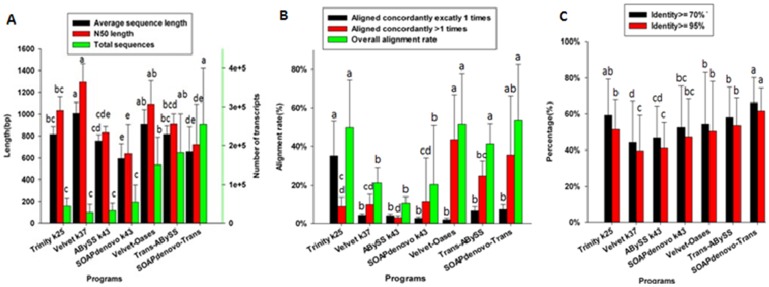
Performance of four programs and SK and MK strategies. Performance measures evaluated included assembly descriptive statistics (A), RMBT percentage (B) and match with wheat reference genes (C). Lower case letters indicate significant differences (at 5% level) among the means for the different programs of the same-colored bars.

Comprehensive comparison showed that SOAPdenovo and ABySS achieved the best results at k  =  43, with more and longer transcripts, whereas Velvet produced better results at k  =  37. Therefore, we compared the assemblies at these k values with the Trinity assembly at k  =  25. The comparison revealed that there were no significant differences among the four programs with respect to total number of sequences. Trinity produced relatively longer transcripts. However, compared with the SK method, the MK method could significantly increase the total number of sequences (Figure 4A, Tables 4 and S5). Trinity assembly (SK) obtained fewer and relatively longer transcripts to other three programs MK assembly (Figure 4A, Table S5). RMBT was an important benchmark for evaluating the performance of each assembler. Theoretically, the optimal program would have the highest RMBT percentage. We found that the RMBT percentage of SK and MK (except for Trinity) assemblies of the four programs varied considerably. The proportion of ACE1 varied from 2.67% (SOAP-Trans, k  =  43) to 35.21% (Trinity, K  =  25), the proportion of AC>1 varied between 3.06% (ABySS, k  =  43) and 11.53% (SOAPdenovo, k  =  43), and the OAR varied from 10.63% (ABySS, k  =  43) to 49.95% (Trinity, K  =  25). Although the MK assemblies produced more transcripts than SK methods, the proportion of ACE1 was not significantly higher (P-value >0.05). The proportion of AC>1 in the MK assemblies and the OAR were significantly higher than that in the SK assemblies (Figure 4B, Table S6). The proportions [of AC>1] were 24.67% for Trans-ABySS MK, 43.49% for Velvet-Oases MK and 35.58% for SOAPdenovo-Trans MK (Figure 4B, Tables 4 and S6). MK methods generated more transcripts and better alignment proportions than SK methods (Figure 4B, Table S6), similar to previous studies [22], [25]. Comparison the Trinity(SK methods) assembly to other three programs (MK methods) indicated that no obvious differences for OAR. However, Trinity assembly was significantly higher in ACE1 and lower in AC>1. The results suggested more redundance in the other programs assemblies (MK methods) (Figure 4B, Table S6). The perfect alignment achieved by Trinity may be attributable to its conservative k-mer-based assembly approach. Trinity had a consistently better performance in all cases than other programs, especially in ACE1 and OAR, while the Trans-ABySS k43 was the worst in OAR

**Table 4:** Evaluation of quality of assemblies from the four programs by BLAST alignment with wheat reference genes.

	Completeness			Contiguity			Accuracy		
Programs	Mean	Sd	Sig 5%*	Mean	Sd	Sig 5%*	Mean	Sd	Sig 5%*
TriFLDB-6137									
Trinity k25	36.99%	18.42%	a	27.71%	12.61%	ab	32.65%	10.20%	cd
Velvet k37	25.14%	19.30%	b	19.76%	18.92%	abc	36.11%	11.13%	c
ABySS k43	14.01%	8.23%	c	7.50%	2.44%	c	52.47%	2.38%	a
SOAPdenovo k43	14.51%	23.79%	c	15.67%	26.13%	bc	33.02%	9.13%	cd
Velvet-Oases	34.76%	20.10%	a	31.77%	18.21%	a	30.01%	6.88%	d
Trans-ABySS	38.78%	18.92%	a	24.89%	10.12%	ab	44.43%	2.72%	b
SOAPdenovo-Trans	42.26%	24.06%	a	15.04%	28.94%	bc	23.89%	7.89%	e
TriallCDNA									
Trinity k25	33.16%	15.26%	ab	25.88%	12.19%	a	37.44%	12.84%	cd
Velvet k37	21.55%	16.17%	c	18.79%	14.19%	ab	42.99%	15.53%	bc
ABySS k43	15.16%	9.27%	d	10.65%	5.34%	b	54.66%	5.22%	a
SOAPdenovo k43	14.89%	20.11%	d	15.07%	21.05%	b	35.70%	9.40%	d
Velvet-Oases	29.59%	16.82%	b	27.32%	15.99%	a	33.39%	9.74%	d
Trans-ABySS	34.18%	16.09%	ab	27.56%	12.63%	a	47.03%	5.30%	b
SOAPdenovo-Trans	38.50%	19.97%	a	13.08%	25.28%	b	33.65%	10.46%	d

Assembled contigs and reference sequences <300bp were excluded from all assemblies.

% level. Different lower case letters within this column indicate that the means were significantly different at the 5

### Program capability in ideal transcript assembly

Although assembly statistics and RMBT metrics could evaluate the performance of programs, the assessment was incomplete without support of reference sequences. Seven assemblies from the four programs were mapped against wheat reference sequence databases (TriFLDB-6137 and TriallCDNA) using BLAST to assess the assembly completeness, contiguity and accuracy. Two-way ANOVA indicated a significant improvement in the completeness and contiguity when the MK strategy was applied to each program (except for SOAPdenovo and Trinity). Compared with the SK strategy, the MK approach produced more total sequences, but the accuracy was not enhanced, especially in SOAP-Trans. For the SK assemblies, Trinity had the best performance in terms of completeness and contiguity. The Trans-ABySS assembly had the highest accuracy rate. However, there was very little difference in accuracy among the assembly results of Trinity (k  =  25), SOAP-Trans (k  =  43), and Velvet-Oases (k  =  37) (Figure 4, Table 4 and Table S7).

**Figure 5:**
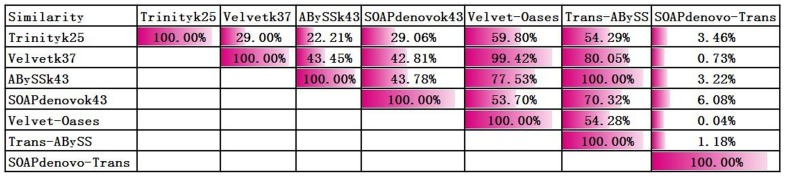
Similarity among the seven assemblies. Pairwise comparison among seven assemblies. Row and column intersections indicated that the two assemblies were more similar.

To assess the detection capability for novel transcripts of the four programs, seven assemblies were aligned to TriallCDNA using BLAST. The highest numbers of reference sequences were detected by Trinity (k  =  25) and SOAPdenovo (k  =  43) (Figure 4C). The percentages of alignments with identity >95% were 59.18% for Trinity (k  =  25) and 52.57% for SOAPdenovo (k  =  43). The MK strategy detected more reference sequences than the SK strategy for the same program. However, the relatively increased number was very small compared to total assembly increased many times generated by MK method (Figure 4C, Table S7). Trinity (SK methods) assembly could detected more references than other three programs. Similarity analysis indicated that there were large differences among the seven assemblies. The Trans-ABySS assembly had the most similarity with the other six assemblies, whereas the SOAPdenovo-Trans assembly had the least overlap. The results suggested that the Trans-ABySS program had the most reliable performance (Figure 5, Table S8). Take together, compared other three programs MK method to Trinity assembly, the Trinity could get relative fewer and lower redundancy transcripts. But these transcripts with significantly higher ACE1, lower AC>1 and more identity with reference sequences.

### Different data volume assembly strategies

**Figure 6:**
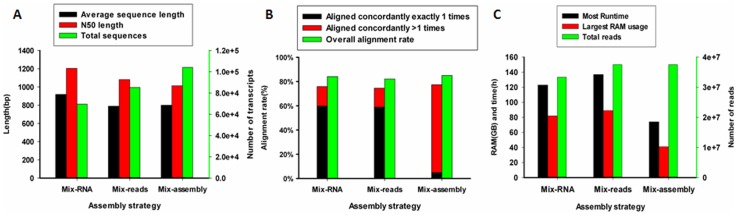
Performance of three assemble strategies. The performance was evaluated by (A) assembly descriptive statistics, (B) RMBT proportion, and (C) longest runtime and largest RAM usage.

Analysis of assemblies constructed from different amounts and variety of starting sequence data showed that, compared with the Mix-RNA strategy, the total reads resulting from the Mix-reads and Mix-assembly approaches were 12.54%, with 4179868 reads, however, the total numbers of contigs of the assemblies were 22.46% (15636) and 49.6% (34532), respectively. Although the total number of contigs was increased, the average and N50 lengths for the Mix-assembly approach were less than for the Mix-RNA approach. When the Mix-HQ-reads and Mix-EDAP-reads were mapped back to the assemblies from the three approaches (Mix-RNA, Mix-read, Mix-assembly), there was little difference in the OAR among the three assemblies (Figure 6, Tables S9 and S10). However, compared with the other two strategies, the Mix-assembly strategy assembled a larger number of contigs. The percentage of AC>1 was high for this strategy and ACE1 was very low, indicating that there was a high degree of redundancy. There was little difference in the mapping metrics between the Mix-RNA and Mix-reads assemblies, but the ACE1 percentage and OAR were increased relative to the Mix-assembly approach, indicating that the Mix-RNA strategy could obtain ideal transcripts. The RAM requirement and runtime for the Mix-RNA and Mix-reads strategies were similar, but the Mix-assembly approach used the least amount of RAM and runtime (Figure 6C). When the Mix-EDAP-reads were used for mapping, the results were very similar, but the proportion of alignment was greatly reduced (Figure 6, Table S10 and S11).

### Hybrid assembly with wheat cDNA

**Figure 7:**
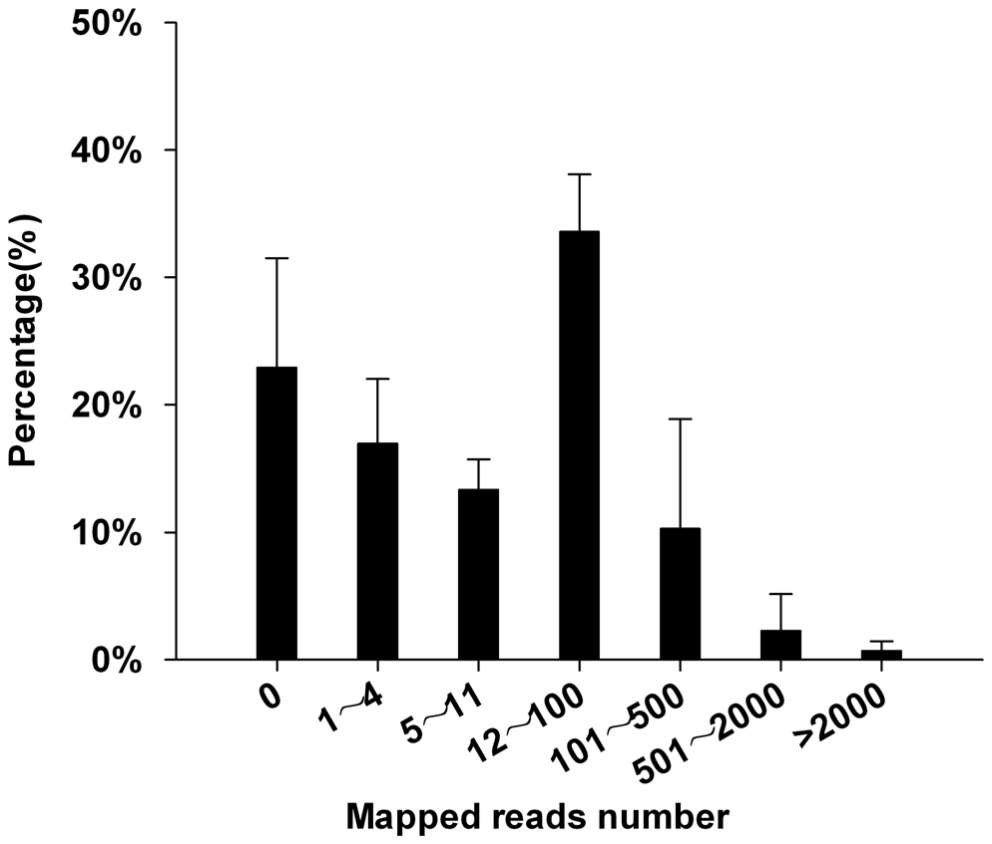
Distribution of HQ reads mapped to TriallCDNA. HQ reads from the four libraries were aligned to the TriallCDNA reference set. Shown is the percentage of mapped reads (y axis) vs. copies (x axis).

TriallCDNA represents nearly all of the wheat genes [4]. To assess the effect of incorporating TriallCDNA into the wheat transcriptome assembly, HQ reads from the four libraries were mapped to TriallCDNA. The distribution of mapped reads showed that the reads that mapped to the reference <4 times accounted for 39.86% of the total reads (Figure 7, Table S12).

Two hybrid assembly strategies, MUTT and ADTT, were assessed by two-way ANOVA. MUTT generated 81506 assembled sequences with an average length of 1058 bp. ADTT generated 77531 assembled sequences with an average length of 1030 bp (Table 5). In contrast, *de novo* assembly generated 40519 sequences with an average length of 816 bp, indicating that the hybrid strategies produce more and longer contigs. RMBT results showed that the MUTT and ADTT strategies had significantly lower proportions of ACE1, higher proportions of AC>1, and higher OARs than the *de novo* assembly. Overall, there were no significant differences between the two hybrid strategies (Table 5).

**Table 5:** Statistics of *de novo*, MUTT, ADTT, CMUTT and CADTT assemblies.

Metrics	*De novo*		MUTT		ADTT		CMUTT		CADTT	
	Mean	Sig 5%*	Mean	Sig 5%*	Mean	Sig 5%*	Mean	Sig 5%*	Mean	Sig 5%*
Number of sequences	45019	c	58537	b	64926	b	75526	a	80,495	a
Average length	816	b	989	a	1011	a	1059	a	1061	a
N50 length	1035	b	1279	a	1330	a	1348	a	1369	a
AC≥1^a^	44.20%	b	47.64%	a	48.51%	a	50.02%	a	50.44%	a
OAR^b^	49.95%	b	53.29%	a	53.88%	a	54.79%	a	55.11%	a

≥1 time is a combination of “ACE1” and “AC>1”.^a^ Aligned concordantly

^b^ Overall alignment rate.

% level. Different lower case letters within this column indicates that the means were significantly different at the 5

### Combined assembly with the wheat A- and D-genome progenitors

Combined strategies were applied to assemble reads from the four libraries using the draft genomes of the wheat A-genome progenitor *T. urartu* and D-genome progenitor *A.tauschii*as references. Two-way ANOVA indicated that all of the assessment metrics were significantly higher for the combined strategies (CMUTT and CADTT) compared to the *de novo* assembly. The total number of sequences increased from 45,019 for the *de novo* assembly to 75526 (CMUTT) and 80495 (CADTT). The average contigs and N50 lengths were increased >200bp and the RMBT increased by an average of 5% (Table 5 and S13). Compared to the MUTT and ADTT strategies, CMUTT and CADTT increased the number of contigs significantly, whereas the other metrics, including length and RMBT, were not obviously improved.

### Final transcriptome assembly and annotation

A final comprehensive transcriptome was generated using what this study determined to be best program (Trinity) and the best strategy (CADTT) for our data. A total of 127083 sequences were generated with an average length of 991 bp and N50 length of 1353 bp. BLAST alignment to NR database showed that 90935(∼71.56%) of the assembled contigs were annotated (Table S14).

## Discussion

### Q30 quality control is necessary

In general, RNA-seq data contains large numbers of low quality reads, which originated during library preparation and the sequencing process. Although variation in the amount of DNA contamination, in the composition of sequencing kits, and in PCR parameters can induce sequencing errors, PCR is the most likely to produce bias [41]. Therefore, maximal initial mRNA amounts and minimal PCR cycle numbers were used to reduce potential bias in the present study. Poor quality reads could lead to fragmented assemblies or false transcripts. Therefore, filtering low quality reads pre-assembly could improve the accuracy and the length of contigs. Conventional quality control threshold are Q13 or Q20, however, comprehensive comparison showed that use of a more stringent quality control threshold (Q30) filtered a large number of sequences, but greatly improved the assembly quality.

The GC content of the raw and HQ reads in this study ranged from 41.83 to 53.1% and from 41.22 to 52.63%, respectively. The difference suggested that there may be a bias in coverage between the different libraries (Table 1). The bias could be introduced at several stages of the standard Illumina sequencing process. For example, high cluster densities in the Illumina flow-cell can suppress GC-rich reads [41]. However, this bias gave us the opportunity to study the effect of GC content on assembly quality, and we found that low GC content in the library leads to more and shorter assembled sequences (EDAP4; Tables 1 and S1). The GC-content bias describes the dependence of read coverage on sequencing data [42]. Previous studies have found that reducing GC-content bias leads to more accurate estimates of fold changes in expression [43]. The present study revealed that Q30 filtering could reduce the GC bias and improve assembly quality (Table S1).

### Program performance

Trinity, SOAPdenovo-Trans, Trans-ABySS and Velvet-Oases were developed specifically for RNA-seq assembly, and have been applied successfully in many previous studies [10], [19], [23], [24], [44]. Other recent studies have compared the performance of these four assemblers using both SK and MK methods [22], [25]. However, test data for this comparison was from species with relatively small, simple, and well-characterized genomes (*Drosophila melanogaster*, *Shizosaccharomyces pombe*, and the tea plant, *Camellia* s*inensis*). The length of *D. melanogaster* genome is ∼122Mb with ∼17,000 genes. The length of genome of *S. pombe* is ∼12Mb with ∼5000 genes. The length of diploid *C. sinensis* genome is ∼4 Gb [45]. Therefore, the conclusion from these previous assembler assessments may not apply to all situations, especially to wheat, with its relatively large (17Gb), complex allohexaploid genome and abundance of repetitive elements. Duan et al. **(**2012**)** optimized *de novo* assembly of a common wheat transcriptome from RNA-seq data. However, this work focused only on the Trinity (SK) and Trans-ABySS (MK) programs. The lack of comparison to other programs, and consideration of hardware requirements, and statistical analysis of results in that study maybe limited for the reliability of their program select in.

The statistical analyses in the present study indicated that the quality of transcriptome assembly was highly dependent on the user-defined k-mer length. The lower the k-mer length, the more transcripts were assembled, although k-mer length did not affect transcript length. The optimal k-mer length for a given data set was associated with the sequencing depth, the base call error rate, and the complexity of the organism from which the transcriptome is being constructed. Higher sequencing depth captures more weakly expressed gene. Base call error interrupts contigs, leading to more and shorter transcripts and, consequently, HQ reads generate longer contigs. Genome characteristics need to be carefully considered, especially for a relatively complex genome like wheat. Longer k-mer length would theoretically lead to a more contiguous assembly of highly expressed transcripts [18], but more moderately expressed transcripts would be better assembled with lower k-mer length [20].

The performance of SOAPdenovo-Trans, Velvet-Oases, and Trans-ABySS was far superior to the SK-based versions of these programs, especially in terms of AC>1 and OAR [25]. Trinity outperformed the other three programs using SK methods in all of metrics. Therefore, assembly could be further improved if an MK strategy was applied in Trinity. However, the application was limited due to its long runtime and fixed k-mer length, and the current version of Trinity would be impractical.

Trinity also outperformed the MK assemblies of the other programs. Compared to the three MK assemblies, Trinity (SK) assembled fewer total sequences with lower redundancy and longer transcripts**,** but with nearly equal OAR (Figure 4A,B), similar to what was observed by Duan et al [6]. Performances of other programs with MK strategy were not satisfactory given the number of low quality assembled transcripts and more redundance.

The amount of sequencing data from the Illumina platform is often very large. Velvet-Oases required the most memory, and Trinity required a runtime >20 fold longer than the other programs. Alternatively, the programs Trans-ABySS and SOAPdenovo-Trans required less memory and had shorter runtime. Taken together, our results indicated that the program used has great effect on assembly outcome.

### Data volume may determine assembly strategies

With the rapid decline in the cost of sequencing, a large amount of wheat RNA-seq data from various tissues and developmental stages has become available. To effectively use this data, it is necessary to choose a suitable assembly strategy based on the sequence volume and hardware requirements. An appropriate assembly strategy could improve transcriptome coverage and accuracy, and save time and money.

The Mix-RNA and Mix-read assembly strategies produced good RMBT values, but because of the large amounts of data generated by these strategies, the computing requirements and runtime (weeks or even months) may be prohibitive to many researchers. Mix-RNA improved the detection of less highly expressed transcripts because of the increased sequencing depth and uniformity in transcript level produced by mixing equal amounts of RNA from the different samples. The Mix-read strategy can also improve uniformity of transcripts because it involves a mix of samples from different development stages or tissues. Therefore, both strategies could improve the assembly quality. The strength of the Mix-assembly strategy is that it has relatively low computer hardware requirements and can be performed in less time. However, owing to lower sequencing depth, a large number of transcripts could not be detected or assembled. Greater sequencing depth in the individual libraries could potentially alleviate this issue. Although the test data was from the same experiment and the same species in this study, the strategies had universality and could be extended to species which have smaller differences on evolution, theoretically. Overall, Mix-RNA and Mix-read are the best strategy when computing capabilities are not a limitation, whereas the Mix-assembly strategy maybe more practical for many researchers.

### Improving assembly quality using public databases

In the present study, stringent quality control and an optimized assembly program were applied to reconstruct a wheat grain development transcriptome. However, many genes were not detected or assembled because of insufficient sequencing depth. Supplementing our data with TriallCDNA improved the transcriptome quality, but 53.2% of our HQ reads mapped to TriallCDNA <11 times (Figure 7, Table S12), and therefore it would not, theoretically, be assembled into full-length transcripts. Even though the contig length threshold was set to 300 bp, there were still many (39.86%) reads that were difficult to assemble because they appeared <4 times. Compared with *de novo* assembly, the two hybrid assemblies (MUTT and ADTT) significantly improved the assembly quality in terms of both statistics and RMBT (OAR = 53.9%). In addition, removal of contigs <300 bp allowed more reads to be mapped back to the assemblies. The ideal RMBT metrics for the Mix sample indicated that a credible wheat grain development transcriptome was assembled.

### Combined strategy

Reference-based assembly is the ideal strategy for transcriptome reconstruct. The lack of a well-established full wheat genome sequence makes reference-based assembly impractical. *De novo* assembly of the wheat transcriptome can reconstruct full-length transcripts, detect novel transcripts and achieve reasonable results [6]. However, this methods lack of the genome and gene structure information. Also, it can not distinguish the paralogs and alleles. The draft genomes of the wheat A- and D-genome progenitors provided the opportunity for reference-based assembly [28], [29]. Unfortunately, the reference is still incomplete because the B-genome sequence is not available. Combining reference-based and *de novo* assembly strategies can bring together the advantages of these two complementary approaches and create a more comprehensive transcriptome [16], as we found in this study (with both CMUTT and CADTT). Slight difference between the two combined strategies (CMUTT and CADTT) showed that all of these two strategies could get ideal assmebly. Substantial increase in total assembled sequences and the smaller increase in RMBT indicated that more alleles and paralogous genes were assembled by the combined process.

### Evaluation metrics

It is difficult to comprehensively evaluate assembly quality because assembly statistics tend to emphasize coverage and contig length rather than accuracy. Many criteria for systematically assessing assembly quality have been established [16], [36], but these standards are only applicable to a simple transcriptome. The TriFLDB-6137 data set was a particularly useful reference for estimating the completeness, contiguity and accuracy of our assemblies. For wheat transcriptome assembly, it is necessary to identify orthologous genes in the A, B and D subgenomes, paralogs and alleles. This auxiliary data set (including 51 WRKY transcription factors and 23 orthologous gene groups from the A, B, and D subgenomes) has been used as a reference to assess redundancy parameters [6]. Removing the majority of this redundancy from the primary library improved assembly quality [6]. Therefore, a large well-characterized reference set that includes transcripts of variable length and expression levels, orthologs, paralogs, and multiple alleles needs to be collected to assess assembly chimerism metrics and variant resolution. The current assessment methods and datasets are insufficient for constructing a wheat transcriptome and new evaluation metrics and algorithm are still needed.

In conclusion, deep sequencing and stringent quality control together with the Trinity SK program and CADTT strategy enabled reconstruction of a relatively accurate and essentially complete transcriptome associated with wheat grain development. These studies could provide aguidance strategy for future wheat transcriptome assembly using RNA-seq data.

## Data Access

The sequence data from this study have been submitted to the NCBI Sequence Read Archive (SRA)(http://www.ncbi.nlm.nih.gov/sra), with accession number is SRP029372.

## Supporting Information

Table S1 **Comparison between raw and HQ reads assemblies by paired t-test analysis.**
(XLSX)

Table S2 **SOAPdenovo-Trans assembly.**
(XLSX)

Table S3 **Velvet-Oases assembly.**
(XLSX)

Table S4 **Trans-ABySS assembly.**
(XLSX)

Table S5 **Comparison of assembly quality among the four programs with SK and MK methods.**
(XLSX)

Table S6 **Comparison of four progrmas assemblies with SK and MK methods by mapping.**
(XLSX)

Table S7 **Statistics of four programs assemblies aligned to TriallCDNA.**
(XLSX)

Table S8 **Statistics of similarity among the seven assemblies.**
(XLSX)

Table S9 **Statistics of single library assembly and merged single assembly.**
(XLSX)

Table S10 **Statistics of three strategies assembly.**
(XLSX)

Table S11 **Comparison of three assemble strategies by mapping.**
(XLSX)

Table S12 **Distribution of Mix HQ map to TriallcDNA assemblies.**
(XLSX)

Table S13 **Statistics of MUTT, ADTT, CMUTT and CADTT assemblies.**
(XLSX)

Table S14 **Transcriptome annotation and statistics.**
(XLSX)
